# Intelligence

**Published:** 1809-03

**Authors:** 


### INTELLIGENCE.

At a meeting of the Wernerian Natural History Society of Edinburgh, on the 14th of January, Dr. Thomson read an interesting description and analysis of a particular variety of copper-glance, from North America. At the same meeting, Dr. John Barclay communicated some highly curious observations which he had made on the caudal vertebræ of the great sea snake, which exhibit in their structure some admirable provisions of nature, not hitherto observed in the vertebræ of any other animal. Mr. Patrick Neill read an ample and interesting account of this new animal, collected from different sources, especially from letters of undoubted authority, which he had received from the Orkneys. He stated, however, that, owing to the tempestuous season, the head, fin, sternum, and dorsal vertebræ, promised some weeks ago to the University Museum of Edinburgh, had not yet arrived; but that he had received a note from Gilbert Meason, Esq. on whose estate in Stronsa, the sea-snake was cast, intimating, that they might be expected by the earliest arrivals from Orkney. In the mean time he submitted to the Society the first sketch of a generic character. The name proposed for this new genus was Halsydras, (from ἁλς, the sea, and ὑδρος, a water-snake;) and as it evidently appeared to be the species described by Pontoppidan, in his Natural History of Norway, it was suggested that its specific name should be H. Pontoppidani.

Mr. James Scott, of Dublin, states, that he has found by repeated experiments, that platina possesses, on account of its imperceptible expansion, a great superiority over other materials for making the pendulum spring of watches; but that arsenic must not be employed in consolidating it, as it would then be liable to expansion. When properly drawn it possesses self-sufficient elasticity for any extent of vibration; it coils extremely well, and if placed when coiled on the surface of a flat piece of metal, making one end of the spring fast, and marking exactly the other extremity, not the slightest expansion is visible when heat is applied. Mr. Scott farther remarks, that he has for a considerable time made use of platina for compensation curbs, and considers it as very superior to steel for every instrument of that kind.

Dr.

Dr. Clarke and Mr. Clarke will begin their Spring Course of Lectures on Midwifery, and the Diseases of Women and Children, on Monday, March 20. The Lectures are read every day, at the house of Mr. Clarke, No. 10, Upper John Street, Golden Square, from a quarter past ten o'clock in the morning till a quarter past eleven, for the convenience of students attending the Hospitals.——For particulars apply to Dr. Clarke, No. 1, New Burlington Street; or to Mr. Clarke, as above.

Dr. Ramsbotham will commence his second Spring Course of Lectures on the Science and Practice of Midwifery, at his house, No. 9, Old Jewry, on Monday the 13th of March, 1809, at seven o'clock in the evening.—Two Courses will be given during the Summer Season; the first of which will be commenced the second Monday in May, at ten o'clock in the morning.—Each Subject of these Lectures is elucidated by appropriate Specimens of natural and diseased parts from a celebrated Anatomical Collection.

Dr. Reid's Lectures on the Theory and Practice of Medicine will commence on Wednesday, the 15th of March, at nine o'clock in the morning, and will be continued every morning, Sundays excepted, for six weeks, when the Course will conclude.

Mr. Taunton's Lectures, which have been suspended through a severe indisposition, will be resumed on Monday the 7th of March.

A Society of Physicians in London has been engaged, for some time past, in collecting materials for a new work, to be entitled the Annual Medical Register. They propose to comprise, in one volume, a complete account of the medical literature of the preceding year, together with an historical sketch of the discoveries and improvements in medicine and the collateral sciences; a report of the general state of health and disease in the metropolis; and a brief detail of such miscellaneous occurrences within the same period, as may be deemed worthy of record.

Mr. Saunders, Surgeon of the London Infirmary for curing Diseases of the Eye, and Demonstrator of Anatomy at St. Thomas's Hospital, proposes, in the course of a few months, to publish a Treatise on some select practical points relating to diseases of the eye, and particularly on the nature of the cataract in persons born blind, and the method which he has for a long time pursued, with uniform success, for the cure of such cases at the earliest periods, and even in infancy.

